# Depth of response to primary POMB/ACE chemotherapy predicts survival in poor prognosis germ-cell tumours

**DOI:** 10.1038/s41416-026-03464-4

**Published:** 2026-05-28

**Authors:** Emily Hobbs, Lara Ulrich, Anand Sharma, Gina Sherpa, Sarah Howlett, Alice Maxwell, Dee Short, Ignacio Vazquez, Roshan Agarwal, David Hrouda, Ethna Mannion, Edmund H. Wilkes, Jingky Lozano-Kuehne, Eslam Maher, Gordon Rustin, Terry Tin, Michael Gonzalez, Naveed Sarwar, Michael J. Seckl, Ehsan Ghorani

**Affiliations:** 1 Department of Medical Oncology, Charing Cross Hospital Campus of Imperial College London, London, UK; 2 Department of Medical Oncology, Mount Vernon Hospital, London, UK; 3 Medical Oncology, University Hospitals of Northamptonshire NHS Trust, London, UK; 4 Department of Urology, Charing Cross Hospital Campus of Imperial College London, London, UK; 5 Department of Histopathology, Charing Cross Hospital Campus of Imperial College London, London, UK; 6 Department of North West London Pathology, Charing Cross Hospital Campus of Imperial College London, London, UK; 7 Biostatistics Research Group, Population Health Sciences Institute, Faculty of Medical Sciences, Newcastle University, Newcastle, UK; 8 Department of Surgery and Cancer, Imperial College London, London, UK

**Keywords:** Germ cell tumours, Tumour biomarkers, Germ cell tumours

## Abstract

**Background:**

Most chemotherapy regimens for poor-risk non-seminomatous germ cell tumours (PRGCT) deliver a fixed number of cycles; the role of variable treatment duration aiming for maximal response is unknown. Since the 1970s, we have utilised POMB/ACE chemotherapy for PRGCT, treating until marker normalisation. From mid-1990s, treatment was limited to 7 cycles. We describe outcomes with POMB/ACE for PRCGT, evaluating the association between treatment duration and survival.

**Methods:**

We conducted a retrospective cohort study across two UK tertiary cancer centres, identifying patients treated between 1978–2013. Complete response was defined based on normalisation of elevated tumour markers, absence of viable cancer in resection material and resolution of radiological abnormalities. Regression techniques were used to investigate the relationship between therapy duration and outcomes.

**Results:**

132 PRGCT patients completed POMB/ACE with 69.7% (*n* = 92) alive at 5 years. Number of cycles delivered significantly correlated with recurrence free survival (HR 0.52; 95% CI 0.38–0.71; *p* < 0.001) and complete biochemical response (OR 1.27; 95% CI 1.10–1.49; *p* = 0.0018). POMB/ACE discontinuation amongst patients with responding but not normalised tumour markers was associated with worse survival. Study limitations include the retrospective design.

**Conclusion:**

POMB/ACE is highly active for PRGCT. Treatment until maximal biochemical response is associated with superior survival outcomes and warrants further exploration.

## Introduction

Poor risk non-seminomatous testicular germ cell tumours (PRGCT) carry a high risk of treatment resistance and death [1]. Whilst studies have explored dose-dense, multi-agent, and high-dose regimens compared to standard therapy with bleomycin, etoposide and cisplatin chemotherapy, these have not yielded improvements in overall survival outcomes [2–10].

Whilst the majority of primary chemotherapy regimens are given for a fixed number of cycles determined by tolerability and toxicity, strategies aiming to treat until maximum biochemical response are understudied.

POMB/ACE chemotherapy has been utilised by our centres since the 1970s. The regimen is composed of cisplatin, vincristine, methotrexate, and bleomycin (POMB) alternating every two weeks with dactinomycin, cyclophosphamide, and etoposide (ACE) [11]. The protocol thus employs highly myelosuppressive agents alternating with those that are less so, minimising treatment interruptions and maximising dose intensity. POMB/ACE has efficacy in PRGCT in several small studies and is additionally active in ovarian germ cell tumours [12–14].

Personalised therapy was a defining feature of POMB/ACE at its inception with patients historically treated with a variable number of cycles, aiming for biochemical complete response. However, extended therapy is limited by toxicity including cytopenia, neutropenic sepsis and peripheral neuropathy. With the introduction of new active agents, we adopted a policy of limiting POMB/ACE therapy to seven cycles in the mid-1990s. Patients who were not in complete remission at this point were switched to alternative regimens. This change in practice provides an opportunity to now examine the relationship between duration of initial therapy and patient outcomes.

Here, we report our experience of the largest cohort of patients with PRGCT treated with POMB/ACE, focusing on whether duration of primary chemotherapy is related to patient outcomes. Our finding that treatment to biochemical remission with first line therapy is associated with improved survival is potentially relevant for the management of PRGCT with chemotherapy regimens other than POMB/ACE.

## Materials (patients) and methods

In this retrospective real-world cohort study, we included patients with PRGCT who commenced treatment with POMB/ACE at two UK referral centres: Charing Cross Hospital, and Mount Vernon Hospital, London, between 1978–2013. PRGCT was defined as per 1997 IGCCCG criteria (Supplementary Table 1). Patients treated before this classification system was published were reclassified accordingly. Patients did not routinely undergo evaluation of lung function or renal function assessment by ^51^Cr-EDTA clearance prior to starting therapy.

### Treatments

POMB/ACE chemotherapy is delivered as alternating cycles of POMB and ACE given every two weeks. POMB comprises IV cisplatin, vincristine, methotrexate and bleomycin. ACE comprises IV dactinomycin, cyclophosphamide and etoposide (Supplementary Table 2). Two consecutive cycles of POMB are followed by ACE alternating with POMB with each cycle given every two weeks, 1 cycle is counted as POMB or ACE given separately. Granulocyte colony-stimulating factor (G-CSF) 300–480 mcg was introduced in the early 1990s and given for 4-8 days, 24 hours after each cycle to reduce the risk of neutropenia.

Historically, treatment was given until marker normalisation followed by 12 weeks of consolidation therapy. From the mid-1990s until the mid-2010s, treatment was limited to seven cycles to limit toxicities. Patients who did not achieve a complete remission after seven cycles were offered alternative regimens including high dose chemotherapy (HDCT) with autologous haematopoietic stem cell rescue. To reduce the risk of early treatment-related mortality, patients with bulky disease or organ failure at presentation were initially treated with one or two cycles of low-dose cisplatin (20 mg/m^2^) and etoposide (100 mg/m^2^) daily for two days every week prior to commencing POMB/ACE [15].

Salvage therapies used in the event of POMB/ACE failure are listed in Supplementary Table 3 and included HDCT [16].

### Outcome categorisation

Primary POMB/ACE response was categorised as success or failure. Success was defined as 1. Biochemical complete remission (CR), i.e. normalisation of markers elevated at POMB/ACE initiation within 30 days of the final cycle; 2. Biochemical CR with POMB/ACE over any period without further treatment except surgery recognising that AFP may take longer to normalise due to liver repair and production after chemotherapy; 3. Locally assessed radiological response and no further treatment post POMB/ACE except surgery for patients with residual disease and without marker elevation at treatment initiation. Scans were not reviewed by RECIST criteria in this retrospective cohort. Normal values of hCG and AFP were defined as <5 IU/L and <15 ng/mL respectively. These cut-offs were consistent over the period of study.

For patients who did not achieve a biochemical CR at the point of POMB/ACE discontinuation, marker response was further classified as falling vs. non-falling. Patients in the falling group had greater than 10% decline in at least one elevated marker, comparing samples in a time window of +14 to –30 days around the final cycle. Patients were further classed as incompletely treated whilst responding (ITR) if POMB/ACE was discontinued in the context of falling markers and patients were subsequently treated with other chemotherapy regimens.

Overall survival (OS) was defined as the duration from cycle 1 POMB/ACE initiation until the last follow-up or death. Recurrence free survival was the duration from cycle 1 POMB/ACE initiation until the last follow-up, recurrence or death. Tumour markers and imaging were evaluated as per standard surveillance protocols.

### Statistical analysis

All analyses were performed in the R statistical programming environment (version 3.4.3). OS was censored at five years. Uni- and multivariable survival analysis by Cox regression modelling was done with the survival package, *p*-values were adjusted using the Benjamini-Hochberg method. Kaplan-Meier plots were constructed with the survminer package. Logistic regression was carried out using the glm function of the stats package. To determine eventual biochemical CR rate amongst patients who changed from POMB/ACE to other regimens whilst still responding, compared to those who continued, we carried out a two-proportions z-test and a Cochran-Mantel-Haenszel test to account for differences in the distribution of treatment duration between the two groups.

### Ethical approval

This work was carried out as a National Health Service evaluation project and did not require external ethics review.

## Results

### Study population

We identified 507 patients who commenced treatment with POMB/ACE chemotherapy between 1978 and 2013. 135 met IGCCCG diagnostic criteria for PRGCT (*n* = 97 at Charing Cross Hospital and *n* = 38 at Mount Vernon Hospitals; 92 had previously been reported [13]). Demographics are described in Table 1. The cohort comprised 87 patients (64.4%) with a poor-risk marker profile, 70 (51.9%) with non-pulmonary visceral metastases and 27 (20%) with primary mediastinal disease. We identified 58 patients with bulky disease or organ failure treated with induction low dose cisplatin and etoposide (EP) prior to commencing POMB/ACE, of whom 37 (63.8%) had one and 21 (36.2%) had two induction cycles. Histology was categorised as per the British Testicular Tumour Panel Classification (Supplementary Table 4) which was in use at the time, whilst the currently used WHO classification system has been widely adopted since.

**Table 1:** Demographic features of 135 patients with PRGCT treated with POMB/ACE between 1978-2013.

Variable	*N* (% or range)
Age (years, range)	28 (14–60)
**Primary site (*n*, %)**	
Testis	88 (65.2)
Mediastinal	27 (20)
Retroperitoneal	13 (9.6)
Unknown/other	7 (5.2)
**Metastatic site (*n*, %)**	
Lung	71 (52.6)
Node	47 (34.8)
Liver	46 (34.1)
Brain	14 (10.4)
Bone	10 (7.4)
Other	10 (7.4)
Mediastinum	5 (3.7)
**Metastatic sites (*n*, %)**	
0	26 (19.4)
1	41 (30.6)
2	45 (33.6)
3	16 (11.9)
4	6 (4.5)
**Tumour marker at diagnosis (median, range)**	
hCG	3515 (0–9,004,032)
AFP	242 (0–321,823)
LDH	336 (0–19,996)
**Poor prognosis marker (*n*, %)**	
hCG (> 50 000)	56 (41.5)
AFP (> 10 000)	32 (23.7)
LDH (> 10xULN)	8 (5.93)
**No. of cycles delivered (median, range)**	7 (1–17)

### POMB/ACE is a highly active regimen in patients with PRGCT

**Fig. 1:**
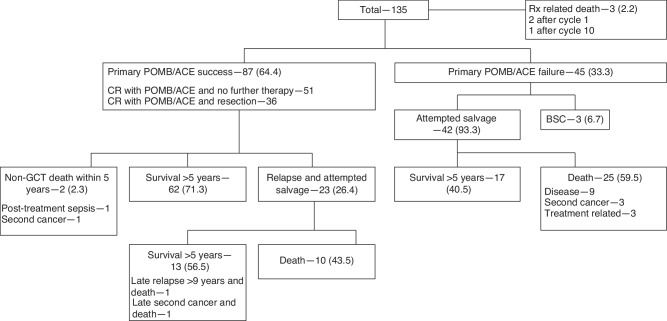
Outcomes after POMB/ACE amongst patients with PRGCT. Number of patients and (percentage) shown. BSC best supportive care.

Patients received a median of seven cycles (range 1–17) of primary POMB/ACE and outcomes are illustrated in Fig. 1. In total, 92 of 132 (69.7%; excluding three deaths on therapy) survived until the five year landmark after treatment initiation. The three patients who died on treatment commenced therapy in the late 1970s. Two deaths occurred after one cycle of treatment in an era before low dose induction EP was introduced. One patient died of sepsis after cycle 10 prior to the introduction of G-CSF support. Including these three deaths, the five year survival is 68.1%.

A total of 87 patients achieved complete remission with POMB/ACE (64.4%) and received a median of seven cycles of treatment (range 4–16). Within this group, 51 (58.6%) were treated with POMB/ACE alone and 36 (40.2%) underwent surgical resection of residual tumour with no active disease identified. Following therapy, 62 (71.3%) remained disease free and survived at least five years, whilst 23 (26.4%) relapsed and were treated with salvage regimens, including four patients (17.4%) who had HDCT. Two patients achieved remission (2.3%) but died within five years; one of post-treatment sepsis and one of a second malignancy.

Most of the relapses after primary therapy (*n* = 19/23) occurred within the first five years (median 10 months). Salvage therapy was successful in thirteen cases (56.5%) who survived at least five years from POMB/ACE initiation and was sustained in most individuals: one patient suffered a late relapse over nine years later and died and another died of a second malignancy.

Amongst 45 patients (33.3%) who failed primary POMB/ACE therapy, 42 had salvage treatments including twelve (28.6%) who had HDCT (Supplementary Table 3). Salvage therapy resulted in sustained remission in 17 patients (40.5%). Amongst those who died within five years, the majority (*n* = 19/25) died of the disease. Six patients died of treatment complications and second malignancy (*n* = 3 in each category).

Due to the multi-decade retrospective nature of the study, toxicity data was only available for 32 (24%) patients, who received a median of 6 cycles of POMB/ACE (range 2-8). Fatigue (59.4%), anaemia (56.2%), vomiting (53.1%) and neutropenia (43.8%) were among the most commonly documented toxicities. The most frequent grade 3 and grade 4 toxicities were thrombocytopenia and neutropenia in 19% and 25% patients respectively (Supplementary Table 5). Retrospective evaluation of toxicity is prone to inaccuracy. As an alternative, we additionally analysed drug dose reductions as an indicator of tolerability (Table 2 and Supplementary Fig. 1). Of 108 patients with available data, 42 (38.9%) had a dose reduction for at least one drug. Cisplatin and methotrexate had the greatest reductions, with median delivered doses of 77% of protocol for 13 and 12 patients, respectively.

**Table 2:** POMB/ACE dose reductions.

Drug	Dose reduction (*n*, %)	Median dose (% of total protocolled dose, range)
Cisplatin	13 (37.1)	77.0 (0–89)
Methotrexate	12 (34.3)	77.5 (31–94)
Etoposide	5 (14.3)	77.5 (0–89)
Cyclophosphamide	2 (5.7)	72.0 (0–89)
Dactinomycin	2 (5.7)	91.5 (89–94)
Vincristine	1 (2.8)	67 (67–67)

For each drug, the table shows the number of patients who had a dose reduction for each drug. Amongst patients who had a dose reduction, for each drug the median dose delivered is represented as a percentage of the protocolled total dose.

### POMB/ACE is active in all patient subgroups and treatment duration predicts survival

**Fig. 2:**
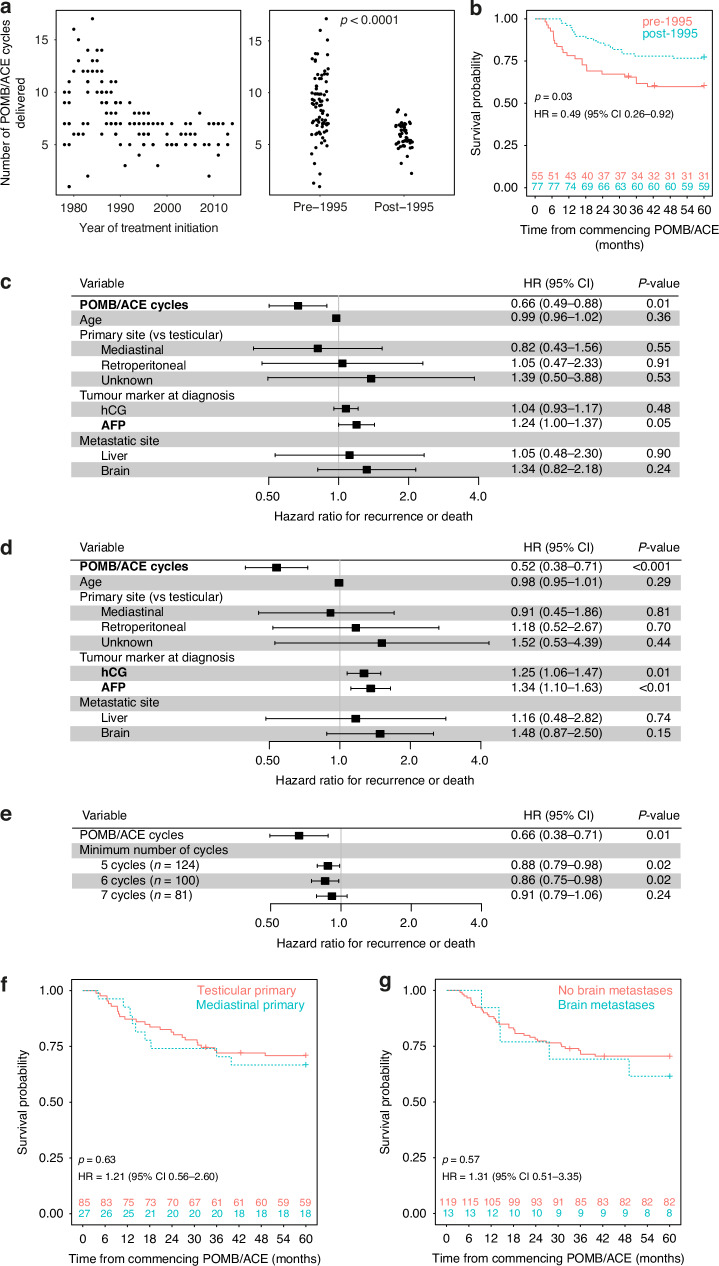
Predictors of outcome amongst patients treated with POMB/ACE. **A** Scatter plots show the change in number of POMB/ACE cycles delivered over time as a continuous variable (left panel) or split into periods before and after 1995 (right panel; *p*-value calculated by two-tailed *T* test). **B** Kaplan-Meier plot shows overall survival in the pre- vs post-1995 treatment eras; Univariable hazard ratio (HR) and *p*-value from Cox regression analysis is shown. Forest plots show the relationship between selected variables and recurrence free survival for 132 patients with PRGCT treated with POMB/ACE in univariable (**C**) and multivariable (**D**) analysis; significant predictors are shown in bold. Calculations were done by Cox regression analysis with *p*-values adjusted for multiple testing. The log10 values of AFP and hCG were used and number of POMB/ACE cycles was analysed in units of three. **E** Forest plot shows the relationship between the number of POMB/ACE cycles delivered and recurrence free survival, with time measured from the final cycle of therapy. Patients who received a minimum of 5, 6 and 7 cycles are shown. **E**, **F** Kaplan-Meier plots show overall survival outcomes for patients with mediastinal vs testicular non-seminomatousGCT (left panel) and brain metastases vs. no brain metastases (right panel). Univariable HRs and *p*-values from Cox regression analysis are shown.

From the mid-1990s, treatment intent was changed from a policy of therapy until maximal biochemical response to one where treatment was capped at 7 cycles, resulting in a reduction in the number of cycles delivered over time (Fig. 2A). Patients treated after 1995 had significantly fewer cycles of POMB/ACE compared to those treated before 1995 (median 9 vs 6 cycles, *p* < 0.0001). Notably, whilst patients treated in these two eras were well matched on relevant baseline characteristics (Table 3), patients treated in the pre-1995 era had a significantly enhanced overall survival (OS; Fig. 2B; hazard ratio [HR] 0.49; 95% confidence interval [CI] 0.26–0.92; *p* = 0.03). We hypothesised this difference in survival suggests a general importance of the number of cycles delivered.

**Table 3:** Clinical and demographic features of patients treated in the pre- and post-1995 eras.

Variable	Pre-1995 (*n* = 80)	Post-1995 (*n* = 55)	*p*-value
**Age (years, range)**	27 (14–57)	30 (18–60)	0.078
**Primary Site (*n*, %)**			0.8
Testis	53 (66.2)	35 (63.6)	
Mediastinal	14 (17.5)	13 (23.6)	
Retroperitoneal	8 (10)	5 (9.1)	
Unknown/Other	5 (6.2)	2 (3.6)	
**Metastatic site (*n*, %)**			0.39
Lung	51 (63.7)	20 (36.4)	
Node	32 (40)	15 (27.3)	
Liver	31 (38.8)	15 (27.3)	
Brain	13 (16.2)	1 (1.8)	
Bone	7 (8.8)	3 (5.5)	
Other	7 (8.8)	3 (5.5)	
Mediastinum	2 (2.5)	3 (5.5)	
**Metastatic sites (*n*, %)**			0.000044
0	5 (6.2)	22 (40)	
1	28 (35)	13 (23.6)	
2	30 (37.5)	15 (27.3)	
3	13 (16.2)	3 (5.5)	
4	4 (5)	2 (3.6)	
**Tumour marker at diagnosis (median, range)**			
AFP	105 (1–80,569)	235 (0–156,058)	0.9
hCG	7877 (1–9,004,032)	269 (0–2,812,057)	0.16
**Poor prognosis marker (***n***, %)**			0.17
AFP	15 (18.8)	16 (29.1)	
hCG	34 (42.5)	18 (32.7)	

To evaluate this, we carried out univariable survival analysis to identify features associated with recurrence free survival (RFS) across the entire cohort and found that the number of POMB/ACE cycles delivered had the strongest association amongst the tested variables (Fig. 2C; HR 0.66; 95% CI 0.49–0.88; *p* = 0.01).

To account for confounding factors, we carried out multivariable Cox regression to correct for the effects of age, baseline tumour marker level, site of disease origin and metastatic site (Fig. 2D). The number of POMB/ACE cycles remained the strongest predictor of outcome in this analysis (HR 0.52, 95% CI [0.38–0.71], *p* < 0.001).

Since survival is measured from the point of POMB/ACE initiation, a longer duration of therapy enriches for patients who live longer. To account for this potential source of immortal time bias, we carried out a landmark analysis. Specifically, we measured RFS from the end of treatment and found that the number of POMB/ACE cycles delivered remained significantly associated with survival (Fig. 2E). We furthermore explored outcomes amongst patients who received a minimum number of cycles ranging from 5–7, since the median number of cycles in the post-1995 era was six. As shown in Fig. 2E, there remained a clear relationship between the number of cycles delivered and survival from treatment completion.

Notably, previously identified poor-prognostic factors including primary mediastinal disease and brain metastases did not predict worse OS outcomes in univariable or multivariable analyses (Fig. 2C, 2D). Analysis of Kaplan-Meier plots of OS confirmed this (Fig. 2F-G), indicating that POMB/ACE is effective across poor-risk subgroups.

#### Treatment duration predicts probability of biochemical remission

POMB/ACE duration has historically been personalised to achieve maximal marker response. We therefore hypothesised that the relationship between duration of treatment and survival is related to enhanced remission rates with longer duration of therapy.

**Fig. 3:**
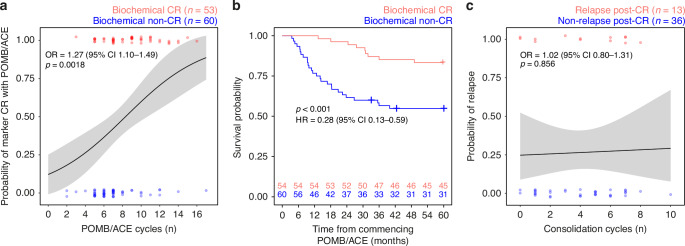
Duration of POMB/ACE therapy and probability of marker complete response. **A** Logistic regression analysis was carried out to evaluate the relationship between number of POMB/ACE cycles delivered and probability of biochemical complete response (CR; red points represent patients who achieved CR, blue points represent patients who did not). **B** Biochemical CR with POMB/ACE associates with overall survival. **C** Logistic regression analysis was carried out to evaluate the relationship between the number of consolidation cycles and probability of relapse amongst patients who achieved marker normalisation with POMB/ACE. OR odd ratio, HR hazard ratio.

To explore this, we classified POMB/ACE response more stringently, defining early marker CR as complete normalisation vs. non-normalisation within 14 days of treatment completion. We next carried out logistic regression analysis to test the relationship between the number of cycles delivered and biochemical response. The number of POMB/ACE cycles was a strong predictor of marker response (Fig. 3A; odds ratio (OR) 1.27, 95% CI [1.10–1.49], *p* = 0.0018). Patients who have a very limited duration of treatment may have stopped because of unacceptable toxicity or rising markers. We therefore restricted the analysis to patients who had five or more cycles and found the relationship still held (OR 1.26, 95% CI [1.09–1.50], *p* = 0.004).

Moreover, patients who achieved biochemical remission had significantly improved survival compared to those who did not (Fig. 3B; HR 0.28; 95% CI [0.13–0.59]; *p* < 0.001). The five-year OS of 83.3% amongst this group supports the notion that achievement of marker CR with first line therapy is highly advantageous.

#### POMB/ACE consolidation therapy does not explain the relationship between treatment duration and outcome

Historically, POMB/ACE was given with the intention to complete 12-weeks consolidation treatment beyond marker normalisation aiming to eradicate marker-negative micro-metastatic disease. Consolidation therapy could thus account for the relationship between POMB/ACE duration, biochemical remission and survival outcomes. However, we found no relationship between the number of POMB/ACE cycles delivered beyond first marker normalisation and relapse probability (Fig. 3C), suggesting extended POMB/ACE consolidation is not beneficial after marker normalisation.

Finally, we found no relationship between the number of POMB/ACE cycles required to achieve marker normalisation (range 2–15) and recurrence free survival (HR 1.04, 95% CI [0.87–1.25], *p* = 0.64). Thus, whilst there is a relationship between treatment duration and outcomes, patients who achieve normalisation later have equivalent outcomes and longer duration of therapy is not associated with survival by virtue of the effects of consolidation therapy.

### Incomplete POMB/ACE therapy is associated with a reduced probability of achieving remission

A further alternative explanation for the relationship between treatment duration and patient outcome is that patients with responsive disease have a greater probability of marker decline and so remain on therapy longer compared to those with treatment resistance and suboptimal marker response.

**Fig. 4:**
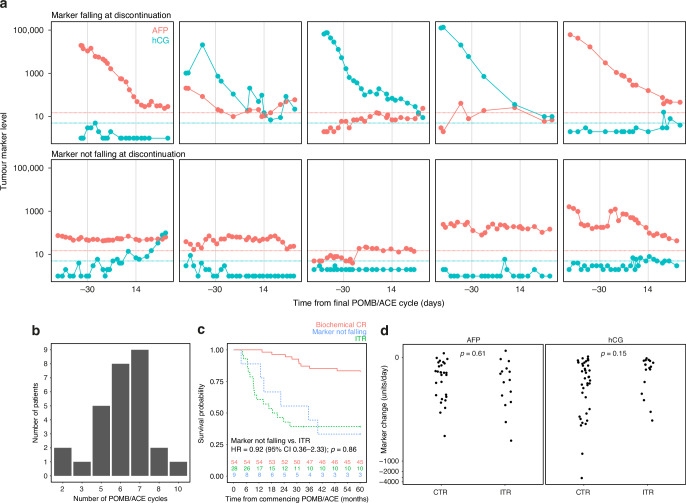
Outcomes amongst incompletely treated responding patients (ITRs). **A** Patients with a falling marker who required further therapy (ITR; top row) or non-falling marker (bottom row) at POMB/ACE discontinuation. Patients with the highest and lowest rates of marker decline are represented. The red horizontal lines represents the threshold for AFP normality, the blue horizontal lines represent the threshold for hCG normality. **B** Cycle at which ITR patients (*n* = 28) discontinued POMB/ACE. **C** Overall survival amongst patients with POMB/ACE biochemical CR, ITR patients and those who stopped POMB/ACE in favour of other treatments with a non-falling marker. **D** The rate of marker change (AFP, left panel; hCG, right panel) is shown for ITR and CTR patients. For CTR patients, marker change was calculated at the last cycle for which markers were falling.

To test this possibility, we studied patients who discontinued therapy with a falling (and therefore responsive) but not normalised tumour markers, as we anticipated more patients would prematurely discontinue therapy in the modern era within which treatment was limited to seven cycles. Tumour marker dynamics for individual patients who stopped vs continued whilst responding are represented in Fig. 4A.

As expected, amongst 48 patients who stopped whilst responding, most were treated after the mid-1990s. The proportion of patients who discontinued POMB/ACE with a falling marker was significantly higher in the more recent era (50.9 vs 25%, *p* = 0.004). Most patients in this category (*n* = 28) were switched to alternative regimens and are defined as incompletely treated whilst responding (ITR) patients. This group mostly stopped POMB/ACE between cycles 5 and 7 (Fig. 4B).

We reasoned that if treatment duration itself increases the probability of achieving remission, ITR patients should have a lower eventual rate of biochemical CR vs. partially treated patients who are responding but continue with POMB/ACE (continued therapy whilst responding; CTR, *n* = 56). Indeed, CTR patients had a significantly greater probability of achieving remission compared to the ITR group (85.7 vs 46.4%, *p* < 0.001). Remarkably, ITR patients had similar OS to those with elevated but not-falling markers at treatment discontinuation due to treatment resistance (Fig. 4C).

Finally, although treatment guidelines do not consider marker dynamics, ITR patients may have switched to other therapies because of a suboptimal rate of marker decline signifying less sensitive disease. To test this, we compared marker decline rates prior to treatment discontinuation between ITR and CTR groups. No significant difference was found (Fig. 4D), indicating that discontinuation in the ITR group was likely a consequence of the protocol-mandated change in maximum duration of therapy rather than due to treatment resistance.

Collectively, these findings suggest a connection between early POMB/ACE discontinuation, reduced probability of biochemical remission and compromised OS independent of disease biology.

## Discussion

We report our several decade experience of POMB/ACE chemotherapy for patients with poor prognosis testicular germ cell tumours. With follow-up data on 135 patients, POMB/ACE remains an effective option with 69.7% of treated patients alive at 5 years. This is comparable to contemporary studies of intensified treatment regimens as shown in Supplementary Table 6 [4, 5, 8, 17]. The overall five-year survival outcome is also in line with contemporary cohorts treated with BEP chemotherapy.

Whilst dose-dense regimens [2–5, 8–11, 18] and consolidation with high-dose chemotherapy [6, 7, 19] have not yielded significant improvements in overall survival outcomes, there is evidence in favour of dose-dense regimens over BEP in specific poor prognostic groups. In particular, those with CNS involvement and primary mediastinal disease may benefit from this approach [1, 8, 17, 20]. However, dose dense regimens are not always effective in this setting as demonstrated by an analysis of the GETUG-13 study [21]. In contrast, patients with brain involvement or primary mediastinal disease treated with POMB/ACE had equivalent outcomes to those without these risk factors in our cohort, indicating this regimen may be an attractive option in this setting. Notably, the GAMEC regimen similarly has been reported to have activity amongst patients with brain metastases [22]. Both POMB/ACE and GAMEC are methotrexate containing regimens and their activity may be related to enhanced CNS penetration [22, 23].

An additional and understudied approach to improving outcomes is personalisation of the duration of chemotherapy, aiming for maximum biochemical response. Uniquely, POMB/ACE was designed to offer a personalised duration of therapy, with treatment until marker normalisation. However, concerns around toxicity and the more recent availability of new active drugs led to a restriction of POMB/ACE duration in favour of switching to other treatments. This change in practice offers an opportunity to study the relationship between treatment duration and patient outcomes for the first time.

We report here that POMB/ACE treatment duration is associated with improved OS. The number of cycles delivered was associated with a higher probability of achieving marker normalisation, which in turn correlates with better survival outcomes; 83.3% of patients who achieved marker normalisation were alive at five years. Treatment beyond marker normalisation was not associated with better outcomes, suggesting the relationship between treatment duration and outcome is not a consequence of consolidation therapy eliminating micrometastatic disease. Notably, patients with falling but not-normalised markers who switched therapy had a significantly lower probability of achieving eventual marker normalisation compared to patients with falling markers who continued POMB/ACE. Patients who prematurely stopped treatment were enriched in the modern era of capping POMB/ACE duration to seven cycles.

The potential benefit of treating to maximal response is supported by clinical and biological data, with numerous studies in solid and haematological malignancies reporting a correlation between depth of response and patient outcomes [24, 25]. Multiple studies of PRGCT have found that the probability of achieving remission in the context of relapsed disease is significantly lower compared to the upfront setting [26–28]. Biologically, relapsed disease may represent an outgrowth of cancer clones that have developed chemoresistance [29]. Evidence that rate of marker decline predicts outcome to first line therapy of PRGCT further supports variable duration treatment, aiming for marker normalisation [7, 9, 30].

A limitation of the current study is its retrospective nature and it is possible that other differences in practice or cohort differences conferred superior outcomes in the pre-1995 era. However, improvements in supportive care, imaging, surgical and salvage interventions would be expected to yield better outcomes over time. Since temporal bias in this retrospective study is challenging to fully address, the concept that extended treatment duration aiming for maximal marker response is beneficial requires prospective evaluation in a multicentre clinical trial.

Toxicities are particularly difficult to identify and grade in the context of historic retrospective studies. With limited data, toxicities were not consistently documented amongst this cohort treated since the late 1970s. This includes toxicities whilst on therapy, late effects and second malignancies. This is a limitation of the study, particularly since our findings suggest the benefits of treatment until maximal response in a relatively young cohort of patients. However, whilst extended therapy with an intense regimen is very likely to increase the toxicity profile, it may also be expected to reduce exposure to HDCT which is itself highly toxic with a 4 - 5% risk of mortality [31, 32] compared to 2% reported with POMB/ACE here. By aiming for maximal response to primary chemotherapy, the number of patients who require HDCT salvage may therefore be reduced.

In summary, we show that POMB/ACE is an effective treatment for patients with PRGCT and is active amongst high-risk subgroups. Treatment duration was associated with a higher probability of achieving marker normalisation and favourable survival outcomes. Patients who switched to other regimens with falling but elevated tumour markers had a lower probability of ever achieving long term remission compared to those who continued with POMB/ACE and our contemporary treatment protocols have reverted back to a policy of therapy to maximal response. Further studies are required to assess the impact of longer treatment duration on acute and long-term toxicity. In general, protocols that personalise the duration of first line chemotherapy for PRGCT may be of significant benefit to patients and further studies to explore this prospectively and in the context of other regimens are warranted.

## Supplementary information

Supplementary Material

## Data Availability

The datasets generated during and/or analysed during the current study are available from the corresponding author on reasonable request.

## References

[1] International Germ Cell Consensus Classification: a prognostic factor-based staging system for metastatic germ cell cancers. International Germ Cell Cancer Collaborative Group. Journal of Clinical Oncology. 1997;15:594–603. 10.1200/JCO.1997.15.2.594. 9053482 10.1200/JCO.1997.15.2.594

[2] Peckham MJ, Barrett A, Liew KH, Horwich A, Robinson B, Dobbs HJ, et al. The treatment of metastatic germ-cell testicular tumours with bleomycin, etoposide and cis-platin (BEP). Br J Cancer. 1983;47:613–9. 10.1038/bjc.1983.99. 6189504 10.1038/bjc.1983.99 PMC2011384

[3] Christian JA, Huddart RA, Norman A, Mason M, Fossa S, Aass N, et al. Intensive Induction Chemotherapy With CBOP/BEP in Patients With Poor Prognosis Germ Cell Tumors. Journal of Clinical Oncology. 2003;21:871–7. 10.1200/JCO.2003.05.155. 12610187 10.1200/JCO.2003.05.155

[4] Cafferty FH, White JD, Shamash J, Hennig I, Stenning SP, Huddart RA. Long-term outcomes with intensive induction chemotherapy (carboplatin, bleomycin, vincristine and cisplatin/bleomycin, etoposide and cisplatin) and standard bleomycin, etoposide and cisplatin in poor prognosis germ cell tumours: A randomised phase II trial (ISRCTN53643604). Eur J Cancer. 2020;127:139–49. 10.1016/j.ejca.2019.12.028. 32007714 10.1016/j.ejca.2019.12.028 PMC7045084

[5] Fizazi K, Pagliaro L, Laplanche A, Fléchon A, Mardiak J, Geoffrois L, et al. Personalised chemotherapy based on tumour marker decline in poor prognosis germ-cell tumours (GETUG 13): a phase 3, multicentre, randomised trial. Lancet Oncol. 2014;15:1442–50. 10.1016/S1470-2045(14)70490-5. 25456363 10.1016/S1470-2045(14)70490-5 PMC4400870

[6] Daugaard G, Skoneczna I, Aass N, De Wit R, De Santis M, Dumez H, et al. A randomized phase III study comparing standard dose BEP with sequential high-dose cisplatin, etoposide, and ifosfamide (VIP) plus stem-cell support in males with poor-prognosis germ-cell cancer. An intergroup study of EORTC, GTCSG, and Grupo Germinal (EORTC 30974). Annals of Oncology. 2011;22:1054–61. 10.1093/annonc/mdq575. 21059637 10.1093/annonc/mdq575 PMC3082158

[7] Motzer RJ, Nichols CJ, Margolin KA, Bacik J, Richardson PG, Vogelzang NJ, et al. Phase III Randomized Trial of Conventional-Dose Chemotherapy With or Without High-Dose Chemotherapy and Autologous Hematopoietic Stem-Cell Rescue As First-Line Treatment for Patients With Poor-Prognosis Metastatic Germ Cell Tumors. Journal of Clinical Oncology. 2007;25:247–56. 10.1200/JCO.2005.05.4528. 17235042 10.1200/JCO.2005.05.4528

[8] Shamash J, Mee M, Sarker S, Wilson P, Ansell W, Greenwood M, et al. Dose-dense chemotherapy for untreated poor-prognosis and relapsed germ-cell tumours: an 18-year experience with GAMEC chemotherapy. BJU Int. 2020;125:843–52. 10.1111/bju.14947. 31688976 10.1111/bju.14947

[9] Fizazi K, Flechon A, Le Teuff G, Mardiak J, Pagliaro LC, Geoffrois L, et al. Mature results of the GETUG 13 phase III trial in poor-prognosis germ-cell tumors (GCT). Journal of Clinical Oncology. 2016;34:4504–4504. 10.1200/JCO.2016.34.15_suppl.4504.

[10] Fizazi K, Le Teuff G, Fléchon A, Pagliaro L, Mardiak J, Geoffrois L, et al. Personalized Chemotherapy on the Basis of Tumor Marker Decline in Poor-Prognosis Germ-Cell Tumors: Updated Analysis of the GETUG-13 Phase III Trial. Journal of Clinical Oncology. 2024;42:3270–6. 10.1200/JCO.23.01960. 39167741 10.1200/JCO.23.01960

[11] Newlands ES, Rustin GJS, Begent RHJ, Parker D, Bagshawe KD. Further advances in the management of malignant teratomas of the testis and other sites. The Lancet. 1983;321:948–51. 10.1016/S0140-6736(83)92079-2. 10.1016/s0140-6736(83)92079-2 6188011

[12] Hitchins R, Newlands E, Smith D, Begent R, Rustin G, Bagshawe K. Long-term outcome in patients with germ cell tumours treated with POMB/ACE chemotherapy: comparison of commonly used classification systems of good and poor prognosis. Br J Cancer. 1989;59:236–42. 10.1038/bjc.1989.48. 2467682 10.1038/bjc.1989.48 PMC2247005

[13] Bower M, Newlands ES, Holden L, Rustin GJS, Begent RHJ. Treatment of men with metastatic non-seminomatous germ cell tumours with cyclical POMB/ACE chemotherapy. Annals of Oncology. 1997;8:477–83. 10.1023/A:1008279222625. 9233528 10.1023/a:1008279222625

[14] Bower M, Fife K, Holden L, Parardinas FJ, Rustin G, Newlands ES. Chemotherapy for ovarian germ cell tumours. Eur J Cancer. 1996;32:593–7. 10.1016/0959-8049(95)00598-6 8695258

[15] Chan Wah Hak C, Coyle C, Kocache A, Short D, Sarwar N, Seckl MJ, et al. Emergency Etoposide-Cisplatin (Em-EP) for patients with germ cell tumours (GCT) and trophoblastic neoplasia (TN). BMC Cancer. 2019;19:770. 10.1186/s12885-019-5968-7. 31382912 10.1186/s12885-019-5968-7 PMC6683367

[16] McNeish IA, Kanfer EJ, Haynes R, Giles C, Harland SJ, Driver D, et al. Paclitaxel-containing high-dose chemotherapy for relapsed or refractory testicular germ cell tumours. Br J Cancer. 2004;90:1169–75. 10.1038/sj.bjc.6601664. 15026797 10.1038/sj.bjc.6601664 PMC2410221

[17] Adra N, Althouse SK, Liu H, Brames MJ, Hanna NH, Einhorn LH, et al. Prognostic factors in patients with poor-risk germ-cell tumors: a retrospective analysis of the Indiana University experience from 1990 to 2014. Annals of Oncology. 2016;27:875–9. 10.1093/annonc/mdw045. 26861605 10.1093/annonc/mdw045 PMC4843188

[18] Culine S, Kramar A, Théodore C, Geoffrois L, Chevreau C, Biron P, et al. Randomized Trial Comparing Bleomycin/Etoposide/Cisplatin With Alternating Cisplatin/Cyclophosphamide/Doxorubicin and Vinblastine/Bleomycin Regimens of Chemotherapy for Patients With Intermediate- and Poor-Risk Metastatic Nonseminomatous Germ Cell Tumors: Genito-Urinary Group of the French Federation of Cancer Centers Trial T93MP. Journal of Clinical Oncology. 2008;26:421–7. 10.1200/JCO.2007.13.8461. 18202419 10.1200/JCO.2007.13.8461

[19] Di Nicola M, Necchi A, Nicolai N, Bengala C, Siena S, Novarino A, et al. 7100 High-dose sequential chemotherapy versus conventional-dose chemotherapy as first-line treatment for advanced poor prognosis germ-cell tumors: a multicenter Phase III Italian trial. European Journal of Cancer Supplements. 2009;7:422.

[20] Feldman DR, Lorch A, Kramar A, Albany C, Einhorn LH, Giannatempo P, et al. Brain Metastases in Patients With Germ Cell Tumors: Prognostic Factors and Treatment Options—An Analysis From the Global Germ Cell Cancer Group. Journal of Clinical Oncology. 2016;34:345–51. 10.1200/JCO.2015.62.7000. 26460295 10.1200/JCO.2015.62.7000 PMC5070579

[21] Loriot Y, Pagliaro L, Fléchon A, Mardiak J, Geoffrois L, Kerbrat P, et al. Patterns of relapse in poor-prognosis germ-cell tumours in the GETUG 13 trial: Implications for assessment of brain metastases. Eur J Cancer. 2017;87:140–6. 10.1016/j.ejca.2017.09.029. 29149760 10.1016/j.ejca.2017.09.029

[22] Hardt A, Krell J, Wilson PD, Harding V, Chowdhury S, Mazhar D, et al. Brain metastases associated with germ cell tumors may be treated with chemotherapy alone. Cancer. 2014;120:1639–46. 10.1002/cncr.28629. 24668504 10.1002/cncr.28629

[23] Smith DB, Rustin GJS, Hitchins R, Bagshawe KD, Hoskins P. Single agent activity of methotrexate in advanced non-seminomatous testicular germ cell tumours. Eur J Cancer Clin Oncol. 1988;24:1779–81. 10.1016/0277-5379(88)90081-8. 2850192 10.1016/0277-5379(88)90081-8

[24] Morgensztern D, Ko A, O’Brien M, Ong TJ, Waqar SN, Socinski MA, et al. Association between depth of response and survival in patients with advanced-stage non–small cell lung cancer treated with first-line chemotherapy. Cancer. 2019;125:2394–9. 10.1002/cncr.32114. 30933354 10.1002/cncr.32114

[25] Kumar S, Paiva B, Anderson KC, Durie B, Landgren O, Moreau P, et al. International Myeloma Working Group consensus criteria for response and minimal residual disease assessment in multiple myeloma. Lancet Oncol. 2016;17:e328–46. 10.1016/S1470-2045(16)30206-6. 27511158 10.1016/S1470-2045(16)30206-6

[26] Zschäbitz S, Distler FA, Krieger B, Wuchter P, Schäfer-Eckart K, Jenzer M, et al. Survival outcomes of patients with germ cell tumors treated with high-dose chemotherapy for refractory or relapsing disease. Oncotarget. 2018;9:22537–45. 10.18632/oncotarget.25162. 29854297 10.18632/oncotarget.25162 PMC5976483

[27] Einhorn LH, Williams SD, Chamness A, Brames MJ, Perkins SM, Abonour R. High-Dose Chemotherapy and Stem-Cell Rescue for Metastatic Germ-Cell Tumors. New England Journal of Medicine. 2007;357:340–8. 10.1056/NEJMoa067749. 17652649 10.1056/NEJMoa067749

[28] Feldman DR, Sheinfeld J, Bajorin DF, Fischer P, Turkula S, Ishill N, et al. TI-CE high-dose chemotherapy for patients with previously treated germ cell tumors: results and prognostic factor analysis. J Clin Oncol. 2010;28:1706–13. 10.1200/JCO.2009.25.1561. 20194867 10.1200/JCO.2009.25.1561 PMC3651604

[29] Taylor-Weiner A, Zack T, O’Donnell E, Guerriero JL, Bernard B, Reddy A, et al. Genomic evolution and chemoresistance in germ-cell tumours. Nature. 2016;540:114–8. 10.1038/nature20596. 27905446 10.1038/nature20596 PMC5553306

[30] Mazumdar M, Bajorin DF, Bacik J, Higgins G, Motzer RJ, Bosl GJ. Predicting Outcome to Chemotherapy in Patients With Germ Cell Tumors: The Value of the Rate of Decline of Human Chorionic Gonadotrophin and Alpha-Fetoprotein During Therapy. Journal of Clinical Oncology. 2001;19:2534–41. 10.1200/JCO.2001.19.9.2534. 11331333 10.1200/JCO.2001.19.9.2534

[31] Weaver C, Schwartzberg L, Hainsworth J, Greco F, Li W, Buckner C, et al. Treatment-related mortality in 1000 consecutive patients receiving high-dose chemotherapy and peripheral blood progenitor cell transplantation in community cancer centers. Bone Marrow Transplant. 1997;19:671–8. 10.1038/sj.bmt.1700713. 9156243 10.1038/sj.bmt.1700713

[32] Kumar L, Ramavath D, Kataria B, Tiwari A, Raj A, Chellapuram SK, et al. High-dose chemotherapy followed by autologous stem cell transplant for multiple myeloma. Indian Journal of Medical Research. 2019;149:730–9. 10.4103/ijmr.IJMR_1593_18. 31496525 10.4103/ijmr.IJMR_1593_18 PMC6755776

