# Supplementary material for: Depth of response to primary POMB/ACE chemotherapy predicts survival in poor prognosis germ-cell tumours
Source: Br J Cancer. 2026 May 28;135(5):708–16. doi: 10.1038/s41416-026-03464-4 (PMC13478161; doi:10.1038/s41416-026-03464-4)
Supplement: Supplementary file 1 [file 41416_2026_3464_MOESM1_ESM.docx]

**Supplementary Figure 1**

**Supplementary Figure 1**. **Dose delivered.** Scatter plot shows the dose delivered for each drug in the POMB/ACE regimen, as a percentage of the protocolled cumulative dose, for 108 patients with available data.

**Supplementary Table 1**

**Poor-Risk NSGCT as per 1997 IGCCCG criteria**

Alpha Fetoprotein (AFP) >10,000 ng/ml

Human chorionic gonadotrophin (hCG) > 50,000 IU/L

Lactate dehydrogenase (LDH) > 10 x ULN

Non-pulmonary visceral metastases

Disease of primary mediastinal origin

**Supplementary Table 1:** Poor-risk NSGST defined by 1997 IGCCCG Criteria, patients should fulfil one or more criteria to be considered poor risk.

**Supplementary Table 2**

**POMB:**

| **Day (s)** | **Drug** | **Dose** | **Administration (route + duration)** | **Diluent/fluid** |
| --- | --- | --- | --- | --- |
| 1 | Vincristine | 1mg/m^2^ (max 2mg) | IV over 10 minutes | 50ml sodium chloride 0.9% |
| 1 | Methotrexate | 300mg/m^2^ | IV over 12 hours | 1L sodium chloride 0.9% |
| 2 | Hydrocortisone | 100mg | IV 30-60 minutes before bleomycin | - |
| 2 | Chlorphenamine | 10mg | IV 30-60 minutes before bleomycin | - |
| 2 | Paracetamol | 1g | PO 30-60 minutes before bleomycin | - |
| 2 | Bleomycin | 15,000 IU | IV over 12 hours | 1L sodium chloride 0.9% |
| 2 | Bleomycin | 15,000 IU | IV over 12 hours | 1L sodium chloride 0.9% |
| 2-4 | Folinic acid | 15mg | PO/IV at 24,36,48,60 hours after start of methotrexate | - |
| 3 | Pre-hydration fluids | - | IV over 2 hours | 1L sodium chloride 0.9% + 20mmol KCL + 4mmol MgSO_4_ |
| 3 | Cisplatin | 40mg/m^2^ | IV over 4 hours | 1L sodium chloride 0.9% + 20mmol KCL |
| 3 | Cisplatin | 40mg/m^2^ | IV over 4 hours | 1L sodium chloride 0.9% + 20mmol KCL |
| 3 | Cisplatin | 40mg/m^2^ | IV over 4 hours | 1L sodium chloride 0.9% + 20mmol KCL |
| 3 | Post-hydration fluids | - | IV over 2 hours | 1L sodium chloride 0.9% + 20mmol KCL + 4mmol MgSO_4_ |
| 4-8 | G-CSF | 300mcg (<80kg)  480mcg (>=80kg) | SC once a day starting more than 24 hours after last chemotherapy | - |

**ACE:**

| **Day** | **Drug** | **Dose** | **Administration (route + duration)** | **Diluent/fluid** |
| --- | --- | --- | --- | --- |
| 1,2,3 | Etoposide | 100mg/m^2^ | IV over 30 minutes | 500ml sodium chloride 0.9% |
| 1,2,3 | Dactinomycin | 0.5mg | IV bolus via side arm of fast running infusion | - |
| 3 | Cyclophosphamide | 500mg/m2 | IV over 30 minutes | 250ml sodium chloride 0.9% |
| 4-8 | G-CSF | 300mcg (<80kg)  480mcg (>=80kg) | SC once a day starting more than 24 hours after last chemotherapy | - |

**Supplementary Table 2: POMB/ACE chemotherapy regimen.** POMB/ACE is an alternating regimen administered every 2 weeks. Treatment starts with two consecutive POMB cycles, then ACE alternates with POMB every 2 weeks. Supportive medications are also included. Granulocyte colony-stimulating factor (G-CSF) 300-480 mcg was introduced in the early 1990s and given for 4-8 days, 24 hours after each cycle. Whilst the POMB requires an inpatient stay over 3-4 nights, the ACE is given daily for 3 days as an outpatient.

**Supplementary Table 3**

| **Regimen** | **Drugs** | **Dose** | **Administration** |
| --- | --- | --- | --- |
| TE/TP (alternates every 2 weeks) | Paclitaxel  Etoposide | 135 mg/m^2^  150 mg/m^2^ | TE given IV day 1 |
|  | Paclitaxel  Cisplatin | 135 mg/m^2^  60 mg/m^2^ | TP given IV day 1 |
| Escalated EP | Etoposide  Cisplatin | 500mg/m^2^  60mg/m^2^ | IV on day 1 |
| TIP | Paclitaxel  Ifosfamide  Cisplatin | 175 mg/m^2^  1000 mg/m^2^  20 mg/m^2^ | IV on day 1  IV day 1-5  IV day 1-5 |
| VIP | Etoposide  Ifosfamide  Cisplatin | 75 mg/m^2^  1200 mg/m^2^  20 mg/m^2^ | IV day 1-5  IV day 1-5  IV day 1-5 |

**Supplementary Table 3:** Common salvage chemotherapy regimens used after POMB/ACE.

**Supplementary Table 4**

| **Histology** | **N (%)** |
| --- | --- |
| Combined tumour (seminoma + TD) | 7 (5.2) |
| Malignant Teratoma Differentiated | 15 (11.1) |
| Malignant Teratoma Indeterminate | 12 (8.9) |
| Malignant Teratoma Trophoblastic | 20 (14.8) |
| Malignant Teratoma Undifferentiated | 27 (20%) |
| Unclassified | 54 (40%) |

**Supplementary Table 4.** Histology of 135 patients with PRGCT treated with POMB/ACE, as per the British Testicular Tumour Panel Classification in use during the period studied

| **Adverse event** | **Total (n,%)** | **Grade 1** | **Grade 2** | **Grade 3** | **Grade 4** | **Ungraded** |
| --- | --- | --- | --- | --- | --- | --- |
| Fatigue | 19 (59.4) | 9 (28.1) | 2 (6.2) |  |  | 8 (25) |
| Anaemia | 18 (56.2) | 1 (3.1) | 12 (37.5) |  |  | 5 (15.6) |
| Vomiting | 17 (53.1) | 5 (15.6) | 6 (18.8) | 3 (9.4) |  | 3 (9.4) |
| Neutropenia | 14 (43.8) |  | 4 (12.5) | 5 (15.6) | 3 (9.4) | 2 (6.2) |
| Thrombocytopenia | 12 (37.5) | 1 (3.1) | 3 (9.4) | 4 (12.5) | 2 (6.2) | 2 (6.2) |
| Peripheral neuropathy | 11 (34.4) | 2 (6.2) | 4 (12.5) |  |  | 5 (15.6) |
| Oral mucositis | 7 (21.9) | 3 (9.4) | 1 (3.1) | 1 (3.1) |  | 2 (6.2) |
| Fevers | 6 (18.8) |  | 3 (9.4) |  |  | 3 (9.4) |
| Constipation | 5 (15.6) | 2 (6.2) | 1 (3.1) |  |  | 2 (6.2) |
| Tinnitus | 5 (15.6) | 2 (6.2) |  | 1 (3.1) |  | 2 (6.2) |
| Chest pain | 4 (12.5) | 1 (3.1) |  | 1 (3.1) |  | 2 (6.2) |
| Diarrhoea | 4 (12.5) |  | 2 (6.2) | 1 (3.1) |  | 1 (3.1) |
| Eye disorder | 4 (12.5) | 3 (9.4) |  |  |  | 1 (3.1) |
| Headache | 4 (12.5) | 1 (3.1) | 1 (3.1) |  |  | 2 (6.2) |
| Dyspnoea | 3 (9.4) | 1 (3.1) |  |  |  | 2 (6.2) |
| Febrile neutropenia | 2 (6.2) |  |  | 2 (6.2) |  |  |
| Oedema | 2 (6.2) | 1 (3.1) |  |  |  | 1 (3.1) |
| Urine infection | 2 (6.2) |  | 2 (6.2) |  |  |  |
| Abdominal pain | 1 (3.1) |  |  | 1 (3.1) |  |  |
| Cellulitis | 1 (3.1) |  |  |  |  | 1 (3.1) |
| Dizziness | 1 (3.1) | 1 (3.1) |  |  |  |  |
| DVT | 1 (3.1) |  | 1 (3.1) |  |  |  |
| Encephalitis | 1 (3.1) |  |  | 1 (3.1) |  |  |
| Line infection | 1 (3.1) |  |  | 1 (3.1) |  |  |
| Neutropenic sepsis | 1 (3.1) |  |  | 1 (3.1) |  |  |
| Pneumonitis | 1 (3.1) |  |  |  |  | 1 (3.1) |
| Rash | 1 (3.1) |  | 1 (3.1) |  |  |  |
| Serositis | 1 (3.1) |  |  |  |  | 1 (3.1) |
| Stroke | 1 (3.1) |  | 1 (3.1) |  |  |  |
| Wound infection | 1 (3.1) |  |  |  |  | 1 (3.1) |

**Supplementary Table 5**

**Supplementary Table 5:** Toxicity data for 32 patients treated with POMB/ACE

**Supplementary Table 6**

| **Study** | **Design** | **Recruitment period** | **N (total)** | **N (PRGCT)** | **Intervention (s) and control (% of PRGCT)** | **PFS/EFS (%; intervention vs control)** | **OS (%; intervention vs control)** | **Toxicity including TRDs** |
| --- | --- | --- | --- | --- | --- | --- | --- | --- |
| Bower et al (1997) | Retrospective cohort | 1977-1995 | 339 | 92 | POMB/ACE (100%)  Median 8 cycles | NR | 75 (3 yr) | 5 early TRDs (hepatotoxicity, pulmonary oedema, sepsis)  5 late TRDs (AML, ALL) |
| Motzer et al (2007) | RCT phase III | 1994-2003 | 219 | 174 | BEP x 2+ HD-CEC x 2 (50%) | 46 (2yr EFS) | 67 (2yr) | 6 TRDs |
|  |  |  |  |  | BEP x 4 (50%) | 45 (2yr EFS) | 69 (2yr) | 4 TRDs |
| Culine et al (2008) | RCT phase III | 1994-2000 | 190 | 115 | CISCA/VB x 4-6 (50%) | 24 (5yr EFS) | 45 (5yr) | 4 TRDs (sepsis)  57% G4 neutropenia |
|  |  |  |  |  | BEP x 4 (50%) | 32 (5yr EFS) | 58 (5yr) | 3 TRDs (respiratory failure)  25% G4 neutropenia |
| Di Nicola et al (2009) | RCT phase III | 1996-2007 | 89 | 89 | BEP x 2 + HD-carboplatin (50%) vs | 56 (2yr EFS) | 61 (5yr) | NR |
|  |  |  |  |  | BEP x 4 (50%) | 59 (2yr EFS) | 67 (5yr) |  |
| Daugaard et al (2011)  EORTC 30974 | RCT phase III | 1999-2007 | 131 | 131 | VIP x 1 + HD-VIP x 3 (50%) | 58 (2yr EFS) | 67 (5yr) | 4 TRDs |
|  |  |  |  |  | BEP x 4 (50%) | 45 (2yr EFS) | 60 (5yr) | 3 TRDs |
| Necchi et al (2014) | RCT phase II | 1996-2007 | 85 | 85 | PEB x 4 (50%) | 55.8 (5yr PFS) | 62.8 (5yr) | 1 TRD |
|  |  |  |  |  | PEB x 2 + HDS (50%) | 54.8 (5yr PFS) | 59.3 (5yr) | 0 TRD |
| Huddart et al (2015)  NCRI TE23 | RCT phase II | 2005-2009 | 89 | 89 | CBOP/BEP x 6 (48%) | 65 (1yr PFS) | 67 (2yr) | 3 TRDs (incl bleomycin lung), 96% G3 haem toxicity |
|  |  |  |  |  | BEP x 4 (52%) | 43 (1yr PFS) | 61 (2yr) | 2 TRDs  63% G3 haem toxicity |
| Fizazi et al (pub 2014, 2016 ASCO) GETUG 13 | RCT phase III | 2003-2012 | 254 | 203 (+UnTMD) | T-BEP-ox x 2 + PIB x 2 (50%) | **60 (5yr PFS)^a^**  *2016 update | 73 (3yr)  *2014 values | 1 TRD  21% G2 neurotoxicity |
|  |  |  |  |  | BEP x 4 (50%) | **47 (5yr PFS)^a^**  *2016 update | 65 (3yr)  *2014 values | 1 TRD  4% G2 neurotoxicity |
| Adra et al (2016) | Retrospective cohort | 1990-2014 | 273 | 273 | BEP x 4 (65%) | 58 (5 yr PFS) | 73 (5 yr) | NR |
|  |  |  |  |  | VIP x 4 (22%) | 57 (5yr PFS) | 74 (5yr) |  |
|  |  |  |  |  | VIP/VB x 4 (9%) | 57 (5yr PFS) | 74 (5yr) |  |
|  |  |  |  |  | BEP x 2 + HD x 2 (4%) | 57 (5yr PFS) | 74 (5yr) |  |
| Kier et al (2017) | Retrospective cohort | 1984-2007 | 1889 | NR | BEP x 4 | 55 (5yr PFS) | 64 (5yr) | NR |
| Shamash et al (2020) | Retrospective cohort | 1997-2016 | 162 | 75  (first line) | GAMEC x 5 | 61.5 (2yr PFS) | 71.9 (3yr) | 5 TRDs  1 case of AML |

**Supplementary Table 6 (overleaf). Summary of trials assessing alternative 1^st^ line chemotherapy vs standard of care (BEP) for poor risk non-seminomatous germ cell tumours (PGCT).** Only trials performed after the IGCCC 1997 criteria for poor risk disease were included to reduce heterogeneity. ^a^Significant difference between arms (HR 0.65, p=0.037); other comparisons in trials with two or more arms not significant. PRGCT, poor risk germ cell tumours; PFS, progression-free survival; EFS, event-free survival; OS, overall survival; TRDs, treatment-related deaths; AML, acute myeloid leukaemia; ALL, acute lymphocytic leukaemia; RCT, randomised controlled trial; TMD, tumour marker decline; UnTMD, unfavourable tumour marker decline; NR, not recorded; PMNSGCT, primary mediastinal non-seminomatous germ cell tumour. BEP, bleomycin, etoposide & cisplatin; POMB/ACE, Cisplatin, vincristine, methotrexate & bleomycin alternating with actinomycin-D, cyclophosphamide & etoposide; HD-CEC, high dose chemotherapy with carboplatin, etoposide & cyclophosphamide; CISCA/VB, cisplatin, doxorubicin & cyclophosphamide alternating with vinblastine & bleomycin; HD-carboplatin, high dose chemotherapy with carboplatin; VIP, etoposide, ifosfamide & cisplatin; HD-VIP, high dose chemotherapy with etoposide, ifosfamide & cisplatin; VIP/VB, etoposide, ifosfamide & cisplatin alternating with vinblastine & bleomycin; T-BEP-ox/PIB, paclitaxel, bleomycin, etoposide, cisplatin and day-10 oxaliplatin alternating with cisplatin, ifosfamide and bleomycin; PEB, cisplatin, etoposide & bleomycin; HDS, high dose sequential chemotherapy; CBOP/BEP, carboplatin, bleomycin, vincristine, cisplatin alternating with bleomycin, etoposide and cisplatin; GAMEC, Granulocyte-colony stimulating factor, actinomycin-D, methotrexate, etoposide, cisplatin.
